# Functional Polymorphisms in *Interleukin-23 Receptor* and Susceptibility to Esophageal Squamous Cell Carcinoma in Chinese Population

**DOI:** 10.1371/journal.pone.0089111

**Published:** 2014-02-28

**Authors:** Bin Ni, Shaomu Chen, Hongya Xie, Haitao Ma

**Affiliations:** Department of Cardiovascular and Thoracic Surgery, The First Affiliated Hospital, Soochow University, SuZhou, China; MOE Key Laboratory of Environment and Health, School of Public Health, Tongji Medical College, Huazhong University of Science and Technology, China

## Abstract

**Background:**

As a key element in the T-helper 17 (Th17) cell-mediated inflammatory process, interleukin-23 receptor (*IL-23R*) plays a crucial role in the pathogenesis of cancer. Single nucleotide polymorphisms (SNPs) in *IL-23R* have been frequently studied in several previous case-control cancer studies, but its association with esophageal squamous cell carcinoma (ESCC) in Chinese population has not been investigated. This study examined whether genetic polymorphisms in *IL-23R* were associated with ESCC susceptibility.

**Methods:**

A hospital-based case-control study of 684 ESCC patients and 1064 healthy controls was performed to assess the association between four previous reported *IL-23R* genotypes (rs6682925, rs6683039, rs1884444 and rs10889677) and ESCC risk. The results revealed that the C allele of the rs10889677A>C polymorphism in the 3′UTR of *IL-23R* gene was inversely associated with the risk of ESCC.

**Results:**

The rs10889677AC genotype had significantly decreased cancer risk (odds ratio [OR] = 0.85, 95% confidence interval [CI] = 0.69–1.01) compared to subjects homozygous carriers of rs10889677AA, the risk decreased even further in those carrying rs10889677CC genotype (OR = 0.64, 95% CI = 0.44–0.93). No significant association was found between the other three polymorphisms and the risk of ESCC.

**Conclusion:**

These findings indicated that rs10889677A>C polymorphism in *IL-23R* may play a protective role in mediating the risk of ESCC.

## Introduction

Esophageal carcinoma is the eighth most common human cancer and the sixth high cancer mortality [1] of which the 5-years survival rate for all stages combined is less than 20% [2]. Esophageal squamous cell carcinoma (SCC) and esophageal adenocarcinoma (EAC) are the two primary types of esophageal carcinoma and they account for more than 95% of all cases of esophageal carcinoma. Some etiologic factors for esophageal carcinoma have been well-established by epidemiological studies including alcohol consumption, cigarette smoking, obesity and dietary factor [3], [4]. However, the fact that a small portion of exposed individuals develop esophageal carcinoma suggests that genetic susceptibility plays a more important role in an individual's risk of esophageal carcinoma.

The interleukin-23 receptor is composed of the IL-23R subunit and the IL-12Rβ1 subunit, and is essential for the Th17 cell-mediated immune response [5]–[7]. Th17 cells are a recently discovered proinflammatory CD4^+^ effector T cell population that contributes to pathogen clearance and tissue inflammation by expressing high levels of the proinflammatory cytokine IL-17 in response to stimulation [8]. Moreover, the novel inflammation pathway axis - IL23/IL17 axis has been shown to play a pivotal role in inflammatory and autoimmune diseases [9]. Thus, IL-23R plays an important role in the initiating, maintaining and accelerating the IL-23/IL-17 inflammatory signal transduction pathway [10]. Moreover, previous studies have indicated that IL-23R can promote tumor growth and may decrease immunosurveillance by CD8^+^ T-cells [11]. These findings suggest that IL-23R may play an important role in cancer development and progression.

IL-23R is encoded by the IL-23R gene, which maps within 151 kb of the IL12RB2 gene on chromosome 1 (1p31.2∼32.1). Several clinically relevant polymorphic sites have been reported in the *IL-23R* gene. However, little is known about the relationship between genetic polymorphisms in the *IL-23R* gene and the susceptibility to ESCC. Furthermore, the microRNA miR-let-7f can block IL-23R expression [12], resulting in the down-regulation of the IL-23/IL-23R pathway and downstream IL-17 production. One of our previous studies have demonstrated that the IL-23R rs10889677A>C SNP may alter *IL-23R* expression by modifying miR-let-7f binding to the 3′UTR of the *IL-23R* gene [13], thereby influence the transcription of *IL-23R in vivo* and *in vitro* in breast, lung and nasopharyngeal cancer. These findings suggest that IL-23R may also play an important role in ESCC development and progression. To test whether the function of these polymorphisms applies to ESCC onset and development, we performed a hospital-based case-control study to investigate the association between various *IL-23R* genotypes and the risk for the development of ESCC.

## Materials and Methods

### Ethics Statement

This study was approved by the medical ethics committee of Soochow University. All the participants were genetically-unrelated ethnic Han Chinese and none had blood transfusion in the last 6 months. Having given a written informed consent, each participant was scheduled for an interview with a structured questionnaire to collect selected information, and to donate 5 ml peripheral blood.

### Study Subjects

The present study of total 684 patients with newly histopathologically diagnosed primary esophageal carcinoma and 1064 sex and age frequency-matched cancer-free controls, who were genetically unrelated ethnic Han Chinese from Jiangsu Province in eastern China. Patients were consecutively recruited from 2001 to 2009, which were diagnosed at The First Affiliate Hospital of Soochow University (Suzhou), with a response rate of 89% [14]. Cancer-free controls were selected from a 3,500 individual nutritional survey conducted in Jiangsu Province in the same period as the cases were collected. The clinical features of the patients are summarized in **Table 1**. There were no age, stage, and histology restriction for cases. The selection criteria for controls included no family history of esophageal cancer and frequency matched to cases on age (±5 years) and sex. Patients or controls that recently (in last 6 months) had blood transfusions were excluded. Having given a written informed consent, each participant was scheduled for an interview with a structured questionnaire to provide data on alcohol use, smoking status, and other factors. This study was approved by the Medical Ethics Committee of Soochow University. At the time of recruitment for the study, informed consent was obtained from each subjects and this study was approved by the Medical Ethics Committee of Soochow University and the Institutional Review Boards of Guangzhou Medical University.

**Table 1:** Distributions of select characteristics among Esophagus carcinoma patients and controls in Chinese populations.

Characteristics	Patients (n = 684)		Controls (n = 1,064)		*P* a
	n	(%)	n	(%)	
**Age (years)**					
≤59	357	52.19	535	50.28	0.465
>59	327	47.81	529	49.72	
**Sex**					
Male	559	81.73	841	79.04	0.19
Female	125	18.27	223	20.96	
**Smoking**					
Ever	391	58.04	522	50.85	0.001
Never	293	41.96	542	49.15	
**Drinking**					
Ever	167	49.56	227	21.33	0.148
Never	517	50.44	837	78.67	
**Body mass index (BMI)**					
≤20	103	15.06	109	10.24	<0.001
20–28	512	74.85	740	69.55	
≥28	69	10.09	215	20.21	
**Stage**					
I	75	10.97			
II	181	26.46			
III	307	44.88			
IV	121	17.69			

a ***P*** **values for a two-sided χ^2^ test**.

### Genotyping Analysis

Genomic DNA was isolated from the peripheral blood lymphocytes of all the subjects. MassArray (Sequenom, SanDiego, CA) was used to genotype all markers using allele-specific MALDI-TOF mass spectrometry for the association analyses as previously described [15]. Primers and multiplex reactions were designed using the RealSNP.com Website. All ESCC patients and healthy controls were genotyped for the rs6682925, rs6683039, rs1884444 and rs10889677 polymorphisms. Eighty samples were randomly selected for direct sequencing to confirm the genotyping results from the mass spectrometric analysis, and the results were in 100% agreement. Approximately 10% of the samples were randomly selected for a blinded repeat of the genotyping without prior knowledge of the previous genotyping result or the status of the case and control. The results of the repeat analyses were in 100% agreement [14], [16].

### Statistical Analysis

Two-sided chi-squared tests were used to assess the differences in the age and gender distributions of the patients in the cancer and control groups as well as differences in the various polymorphisms and the presence of disease. The Hardy-Weinberg equilibrium (HWE) was tested with a goodness-of-fit chi-squared test to compare the expected genotype frequencies with the observed genotype frequencies (*p^2^+2pq+q^2^ = 1*) in the cancer-free controls. The association between the case-control status and each SNP, which was measured by the OR and its corresponding 95%CI, was estimated using an unconditional logistic regression model with adjustments for age, gender, smoking status, drinking status, and BMI as appropriate. Logistic regression modeling was also used for the trend test. Data were further stratified by characters to evaluate the stratum variable-related ORs among the *IL-23R* rs10889677A>C genotypes. Homogeneity among stratum variable-related ORs was tested. The false-positive report probability (FPRP) test was applied to detect the probability of false-positive association findings [17], [18]. All tests were two-sided and were performed with the SAS software (version 9.1; SAS Institute, Cary, NC, USA). A *P*-value<0.05 was considered statistically significant.

## Results

### Identification of *IL-23R* SNPs Associated with ESCC Susceptibility

To test whether *IL-23R* polymorphisms are associated with ESCC risk, we performed the genotypic distribution of four candidate SNPs (rs6683039, rs6682925, rs1884444 and rs10889677) of the *IL-23R* gene between cases and controls. Our case-control analysis included 684 ESCC patients and 1064 healthy controls. The genotype results are summarized in **Table 2**. A significant association with low risk of ESCC was observed for the rs10889677A>C SNP (OR for the rs10889677AC genotype, 0.85; 95%CI: 0.69–1.01; OR for the rs10889677CC genotype, 0.64; 95%CI: 0.44–0.93; *P*_trend_ = 0.004). The other SNPs, rs6682925 and rs6683039 in the promoter region and rs1884444 in exon 2, were not significantly associated with the risk of ESCC in our study population (**Table 2**). Thus, we may conclude that genetic variant rs10889677CC polymorphism in IL-23R plays a significantly protective role in mediating the risk of ESCC.

**Table 2:** Distribution of genotypes of *IL-23R* gene and associations with the risk of Esophagus carcinoma.

Genotype	Patients (684)	Controls (1064)	Adjusted OR^a^ (95% CI)
rs6682925			
TT	251	391	1.00(Reference)
TC	335	490	1.02(0.81–1.29)
CC	98	183	0.73(0.52–1.02)
*P*_trend_			0.409
rs6683039			
TT	240	396	1.00(Reference)
TC	342	483	1.16(0.93–1.44)
CC	102	185	0.91(0.67–1.23)
*P*_trend_			0.92
rs1884444			
TT	350	519	1.00(Reference)
TG	274	431	0.87(0.69–1.10)
GG	60	114	0.80(0.55–1.17)
*P*_trend_			0.182
rs10889677			
AA	360	498	1.00(Reference)
AC	279	462	0.85(0.69–1.01)
CC	45	104	0.64(0.44–0.93)
*P*_trend_			**0.004**

a **Data were calculated by unconditional logistic regression, adjusted for age, sex, BMI, smoking and drinking.**

***P*** **<0.05, the values of which were presented in bold, was defined as statistically significant.**

### Stratification Analysis of IL-23R Rs10889677A>C Genotypes and the Associated Risk of ESCC

A stratification analysis according to by subgroup of age, sex, smoking status, alcohol drinking status, and BMI to further verify the association between the risk of ESCC and *IL-23R* rs10889677A>C genotypes was conducted. As shown in **Table 3**, we observed significant heterogeneity in the genotype distribution between smoking and no smoking patients (*P* = 0.03). Compared with the AA genotype, the C allele carriers (AC+CC) had much more decreased risk for developing ESCC in non-smoking patients (adjusted OR = 0.65, 95%CI = 0.50–0.85) than the smoking subgroup (adjusted OR = 1.00, 95%CI = 0.75–1.33). However, there were no significant heterogeneity in other subgroups.

**Table 3:** Stratification analysis of the *IL-23R* rs10889677A>C genotypes by selected variables in esophageal cancer patients and controls.

	Patients (n = 684)		Controls (n = 1064)		Adjusted OR (95% CI)^a^	*P* value^b^
Characteristics	AC+CC	AA	AC+CC	AA	AC+CC vs. AA	
**Age(years)**						
≤59	166	191	294	241	0.71 (0.54–0.93)	0.27
>59	158	169	272	257	0.88 (0.67–1.16)	
**Sex**						
Male	266	293	462	379	0.74 (0.60–0.92)	0.25
Female	58	67	104	119	0.99 (0.64–1.54)	
**Body mass index**						
≤20	57	46	66	43	0.81 (0.47–1.39)	0.28
20–28	242	270	386	354	0.82 (0.66–1.03)	
>28	25	44	114	101	0.50 (0.29–0.88)	
**Smoking**						
Positive	173	218	287	235	1.00 (0.75–1.33)	**0.03**
Negative	151	142	279	263	0.65 (0.50–0.85)	
**Drinking**						
Positive	78	89	113	114	0.88 (0.59–1.32)	0.55
Negative	246	271	453	384	0.77 (0.62–0.96)	
**Stage**						
I	34	41	566	498	0.73 (0.46–1.17)	0.61
II	94	87	566	498	0.95 (0.69–1.30)	
III	139	168	566	498	0.73 (0.56–0.94)	
IV	57	64	566	498	0.78 (0.54–1.14)	

a **ORs were adjusted for age, sex, BMI, smoking and drinking as appropriate in a logistic regression model.**

b ***P*** **value of the test for homogeneity between stratum-related ORs for** ***IL-23R*** **(rs10889677 AC+CC vs. AA genotypes).** ***P*** **<0.05, the values of which were presented in bold, was defined as statistically significant.**

## Discussion

Associations between ESCC susceptibility and IL-23R polymorphisms have not been detected in any population using cases-controls study. In this molecular epidemiological study we sought to identify genetic factors that confer individual susceptibility to ESCC. Our results obtained by analyzing 684 ESCC patients and 1064 healthy controls showed that the functional variation rs10889677 C in the *IL-23R* was associated with decreased risk for developing ESCC. We also found that the protective effect of this polymorphism was more pronounced in nondrinkers, namely, the high risk of ESCC was more pronounced in alcohol drinking subjects in the stratification analysis. It was generally accepted that ESCC is a complex disease that its etiology is related to environmental exposures, genetic loci and gene-environment interactions, and alcohol drinking was one of major risk factors for ESCC [19]–[21]. Here, our finding also indicated a gene-environment interaction between alcohol use and genetic variation for developing ESCC. For the other three polymorphisms, there exists no significant difference in the susceptibility of ESCC between different genotypes. Combined with our previous study of these polymorphisms in breast, lung, and nasopharyngeal cancers with diverse etiologies [13], our data further raises the possibility that *IL-23R* variant might be a common susceptibility factor for human cancer.

The importance of IL-23R in tumor development and its influence on tumor immunity has been well recognized [22]. Therefore, it is biologically reasonable that functional IL-23R polymorphisms may play a role in the development of cancer. In fact, studies have shown that IL-23R polymorphisms are associated with susceptibility to gastric cancer [23]. Chen et al. found in a previous study of gastric cancer, which included 941 cancer patients and 775 Chinese (Guangzhou) control subjects, that the rs10889677CC genotype is associated with a significantly reduced risk of gastric cancer when compared to the more common rs10889677AA genotype (OR = 0.47, 95% CI = 0.31–0.71) [24]. Other two independent studies of Chinese population have shown that the rs10889677C allele may increase the risk of oral cancer [25] and ovarian cancer [26] when compared to the rs10889677A allele. Nevertheless, these two studies were based on a relatively small number of study subjects (240 oral cancer patients and 240 Taiwanese control subjects; 96 ovarian cancer patients and 115 Chinese control subjects), which provided inadequate statistical power to draw strong conclusions (20.2% and 14.4%, respectively). Recently, several genome-wide association studies (GWAS) have reported several novel SNPs that are associated with the development of breast cancer [27]–[33]. However, most of the studies were based on Caucasians and only one GWAS was based on Chinese. A GWAS of nasopharyngeal carcinoma also reported three susceptibility loci in Chinese population, including rs9510787, rs6774494 and rs1412829 [34]. Interestingly, candidate genes of these loci may relate to the immune response [35]–[37]. The IL-23R gene, which maps to chromosome 1 (1p31.2∼32.1), GWAS have reported that chromosomal loci from 1p31 to 1p36 were strongly associated with the development of cancers in Asian populations [38], [39]. Kiyotani et al. [38] identified locus at 1p31 associated with clinical outcomes of breast cancer patients with tamoxifen treatment in Japanese, and our previous GWAS data in Chinese populations have also indicated that these loci may be associated with lung cancer risk [40]. Due to the limitation of SNP coverage in the Affymetrix Genome-Wide SNP Array 6.0 chip, previous GWAS regarding cancer in Chinese patients do not include these SNPs. therefore, the association between IL-23R rs10889677A>C polymorphism and the risk for ESCC in Chinese population remains unclear, and case-control studies with large sample sizes and different cancer types are needed [15], [41]. Recently, polymorphisms of various genes, both tumor suppressor genes [such as *breast cancer susceptibility gene 1* (*BRCA1*) [20], *P53* [42] and *Flap endonuclease 1 (FEN1)*] [43] and oncogenes (such as *MDM4*) [21], have been determined in multiple cohorts and are associated with the susceptibility to gastrointestinal cancers. In addition, it have been confirmed that polymorphisms determined by GWAS also play important role in Esophagus carcinogenesis [19], [44].

Evidence for the biological function of the IL-23R rs10889677A>C SNP has been reported in previous studies. Our previous study has demonstrated that the IL-23R rs10889677A>C SNP may affect IL-23R expression by modifying miR-let-7f binding to the 3′UTR of the IL-23R gene. miR-let-7f binding affects the expression of IL-23R on the cell surface, thus influencing the IL-23/IL-17 pathway and the immunosuppressive function of Tregs in the tumor environment. Li et al. [12] have shown that miR-let-7f inhibits IL-23R expression in human CD4+ memory T cells, and transfection of memory T-cells with miR-let-7f mimic results in the downregulation of IL-23R and the downstream cytokine IL-17. Previous studies have reported that miR-Let-7f may be a tumor suppressor gene that plays an important role in cancer development [45]. IL-23R signaling in Tregs promote an immunosuppressive phenotype in the tumor environment [46], which promotes carcinogenesis. Tumor-associated Tregs express the IL-23 receptor, which activates Stat3, resulting in the upregulation of the Treg-specific transcription factor Foxp3 and the immunosuppressive cytokine IL-10.

Our results regarding associations between the IL-23R rs10889677A>C polymorphism and susceptibility to ESCC were obtained from a case-control study derived from eastern Han Chinese population. There are several limitations of this study, including its retrospective design and moderate sample size. Results from this study should not be over-interpreted before they are validated by larger prospective population-based studies. The relatively large sample sizes effectively decreased the ORs and increased the significance of our findings. The genotype frequencies of all the SNPs in the controls were aligned with HWE and supporting the random nature of our method for selecting controls. Moreover, the associations between the SNPs and cancer risks observed in our study are biologically plausible and consistent with our functional findings. Nevertheless, previous studies of breast, lung and nasopharyngeal cancers as well as other cancers showed the same tendency of the function of this SNP. In conclusion, our study demonstrated an association between the IL-23R rs10889677C allele and reduced risk of ESCC. Together with previous studies [13], [47], [48], the rs10889677C allele resides in the 3′UTR of the IL-23R gene and results in the inhibition of the interaction with Th17 and Treg cells, which consequentially increases the proliferation rate of T lymphocytes and may explain the observed decrease in ESCC susceptibility.
